# Do Social Pension Schemes Promote the Mental Health of Rural Middle-Aged and Old Residents? Evidence From China

**DOI:** 10.3389/fpubh.2021.710128

**Published:** 2021-07-29

**Authors:** Guochen Pan, Shaobin Li, Zhixiang Geng, Kai Zhan

**Affiliations:** ^1^Department of Insurance and Actuarial Science, Economics and Management School, Wuhan University, Wuhan, China; ^2^Risk Research Center of Wuhan University, Wuhan, China; ^3^Department of Risk Management and Insurance, School of Finance, Guangdong University of Foreign Studies, Guangzhou, China

**Keywords:** urban-rural resident social pension, new rural social pension, depression, mental health, rural residents

## Abstract

As China experiences rapid aging, the mental health of older rural adults has become a major public health concern. Among other social insurance programs, the New Rural Social Pension (NRSP) scheme was established to replace part of the income for old-age rural residents in China. This article employs survey data from the China Health and Retirement Longitudinal Study (CHARLS) in 2015 and 2018 to investigate the impact of a pension on depression in middle-aged and old residents. Our results show that the pension scheme not only reduces the depressive symptoms of the rural residents but keeps down the prevalence rate of depression. Among the subscribers of the pension scheme, the pensioners benefit more from enrolling in the pension scheme than the contributors in terms of depression alleviation. The impact of pension on depression displays heterogeneity; female residents, residents in central China, and/or those from lower income households are found to be positively affected. It is also confirmed that a pension scheme contributes to easing depression via reduced labor supply, better family support, and more consumption expenditure.

**JEL Classification:** H55, I18, I38.

## Introduction

Due to improved medical conditions, higher living standards, and a declining birth rate, accelerated aging around the globe seems to be unavoidable. Among all the major countries, China especially is experiencing a rapid process of population aging. According to the seventh national census of China, the population of those above 60 and 65 reached 264.02 and 190.64 million in 2020, taking up 18.7 and 13.5% of the total population, respectively. The proportion of the aged in the total population is expected to surge in the coming decades.

What makes the situation worse are the significant socioeconomic changes accompanying rapid economic growth in China. China used to be a dichotomous society where the urban and rural areas were different in many aspects, such as income level, social support systems, etc. (1). When industrialization and urbanization accelerated, the dichotomy was exacerbated. A lot of labor force moved into the cities, and villages were seen “hollowing out,” where only the elderly and children were left behind. Without a proper social support system, this population was vulnerable to mental health problems such as depression. Accordingly, some researchers noticed that depression is more prevalent in rural areas than cities (2, 3). It was reported that the prevalence rates of depression in urban and rural areas were 16.4 and 30.0% in China in 2013, respectively (4). According to WHO (5), among all the victims of depression, the undertreated cases for rural residents were twice as many as among urban citizens.

Depression was found to be closely associated with numerous undesirable consequences (6). It also resulted in heavy medical burden on the family and society. According to Fan et al. (7), mental diseases like depression accounted for over 20% of the total medical expenses in China. Thus, depression has already become a major public health concern.

Correspondingly, much research has been carried out to identify the determinants of depression. Factors which could have a significant impact on depression can generally be classified into several groups. The first category of determinants are demographic characteristics, such as gender, age, education level, and income level (8–11) find that Chinese women have significantly higher depressive symptoms than men. Socioeconomic status (SES) is also identified as a risk factor for depression: people with lower SES generally show higher degrees of depressive symptoms (12, 13). The second category of determinants involves health conditions, such as functional disability and chronic diseases (14). The third group of determinants are environmental factors, such as living facilities and social support from community or family (15, 16). Fang et al. (1) also attributes the worse mental health of rural residents to limited access to community support. The fourth group of factors are related to social participation. Higher frequency of participation in social activities and diversity of social activities involved are positively related with better mental health (16, 17). Active participation in social activities is beneficial for improving mental health, such as reducing depressive symptoms, and improving life satisfaction (18–22). Aside from above factors, the impact of government welfare provisions has attracted the attention of researchers. Calvo (23) believed that benefits provided by the government can improve life satisfaction of citizens significantly. Kim (24) also reported similar impacts of public social safety nets on the life satisfaction of elderly Korean citizens. Provision of better-quality pension schemes was found to improve the life satisfaction of employees both during and prior to retirement in China (25). Grogan and Summerfield (26) found that pension benefits reduced the labor supply of retired female pension subscribers and increased their happiness. Enrollment in pension schemes could help older adults reduce their anxiety and loneliness and promote their self-care capability and life satisfaction (27–29).

In 2009, the Chinese government launched a new social pension scheme for rural residents. This program was designed to provide income for old-age rural residents, but it may bring other effects as well. Specifically, its impact on elder residents' depression needs to be examined. If it is found to positively impact mental health, the pension scheme should be further consolidated to strengthen the function.

Even though most studies supported a positive correlation between pension scheme and mental health (27, 28, 30), there were still many questions left unanswered. Firstly, nearly all the literature assumed that the effect of pension on mental health is the same irrelevant of the state of pension, i.e., as a contributor or pensioner (31), but this assumption could be challenged, because the impact of paying premiums and receiving proceeds won't be the same for an ordinary person, even if this person is considered as “under coverage.” Secondly, the effects of various covariates were usually found to be inconsistent with each other in literature; more empirical evidence was needed. Thirdly, the mechanism of the effect of pension enrollment on mental health was still like a “black box”; some functioning channels were identified (27), (32), (33), but it was far from being exhausted, meaning those identified functioning channels need to be further tested. Last but not least, most previous studies on this topic were carried out with small samples (34). Evidence from large samples is earnestly expected.

Using a national survey database and a 10-questions version of the Center for Epidemiologic Study depression (CES-D) battery to gauge the extent of depressive symptoms, this article confirmed that the subscribers of the pension scheme, especially the pensioners who are eligible to receive benefit but do not need to contribute any longer, have fewer depressive symptoms and less incidence of depression than the contributors who are not eligible to receive benefit yet but suffer from the burden of paying the premiums. This effect is heterogeneous among populations with different characteristics, and work through some mediators, such as labor supply, family support, and consumption expenditure.

This article is organized as follows: section 2 presents a brief introduction about the background of rural social pension scheme in China, section 3 introduces the methodology and data, section 4 displays the empirical results, section 5 identifies the mechanism of the effect of pension, and section 6 gives the conclusive remarks.

## Background of Rural Pension Scheme in China

After several failed trials, the State Council of China initiated a new round of rural pension system reform and established a new scheme called the New Rural Social Pension (NRSP) scheme in 2009. According to the design of the scheme, the pension fund will be contributed to by the subscriber, the employer, and the local and central government. The reform was first launched in 320 pilot counties; afterward, the scheme was extended to all the counties around the country by the end of 2012. NRSP was incorporated into an urban residents' pension scheme and renamed as Urban-Rural Resident Social Pension (URRSP) in 2014^1^. According to NRSP, every rural resident who was beyond 16 and had not been enrolled in any other pension plan was qualified to join NRSP. When NRSP was initiated at the local area level, if the rural subscriber was beyond 60, he/she was entitled to receive basic pension benefit from the fund and pension premium was no longer required, on the condition that the qualified children of the subscriber shall be subscribed in the scheme. When an individual was <15 years from the pension age, the pension premiums should be paid on an annual basis, at the same time, a supplementary premium payment was allowed to make the total premiums payment be no <15 years; when an individual was more than 15 years from the pension age, the pension premiums should be paid on an annual basis, and the premiums should be paid for more than 15 years. The individual payment standard was classified into 100, 200, 300, 400, and 500 yuan per year. The subscribers had the option to choose one standard which suited them; if they chose to contribute more, they would benefit more. The village and other social entities were allowed to subsidize the individual payment of premiums on a voluntary basis. Local government was required to subsidize the subscriber for no <30 yuan per year from the public finance account. There were two accounts for the NRSP, namely individual account and social pooling account. All the individual premiums would go into an individual account and accumulate, the subscriber could draw benefit from it according to a computation formula in their old age. When the subscriber was deceased, the remainder in the individual account could be inherited. Social pooling account was fully financed by the local or central governments, and the funds were used to pay the basic benefit of pension. The standard of basic benefit was set at 55 yuan per person per month (or 660 yuan per year). The central government promised to finance all the funds in the social pooling account for central or western provinces, and half of the funds for relatively developed eastern provinces. Since 2020, some reforms about the URRSP have been carried out to encourage individuals to contribute more; the specific policy provisions include lifting the premium payment ceiling and more standards of premiums.

Compared with developed countries, the pension benefit is inappreciable in its amount. But the significance of the pension benefit should be better evaluated by its share in the net income of the subscribers. When the share is relatively high, the subscribers will have a stronger feeling of been protected financially, and their worries about their financial situation in old age will be greatly reduced. According to a survey in 2009 when the policy was formulated, the 660 yuan basic benefit of pension took up 8–30% of the net income for the rural old adults ranging 60–75. The average was about 15%, and the pension benefit became more and more important as the total net income declined with old age.

## Data and Methodology

### Source of Data

Even though the issue of depression in China has been investigated in the literature, most previous studies employed regional data which only reflected the situation in a specific area, and usually the volume of data was small. This article employs data from a large-scale national survey, the China Health and Retirement Longitudinal Study (CHARLS), to conduct the research. The CHARLS survey was directed by the National Development Academy of Peking University, and aimed to investigate older adults above 45 and their families. The survey was initiated in 2011, then three follow-up surveys were carried out in 2013, 2015, and 2018. Due to the scientific sampling methods and the large volume of sample size, CHARLS data was widely acknowledged and used in many previous literature to examine the health status of older adults in China, such as in Lafave (35) and Lei et al. (36). Considering the NRSP was declared by the government to have spread to each and every county in China at the end of 2012, and to make sure all the respondents were affected by the policy, we drop the data of 2011 and 2013 and only use those of 2015 and 2018 to constitute panel data sets. After deleting data with missing information, 21,122 samples are included in our study. Data and documentation of CHARLS are available at http://charls.pku.edu.cn/.

### Variables

#### Dependent Variable

The dependent variables included in our research are the extent of depressive symptoms and the incidence rate of depression. The Center for Epidemiological Study Depression Scale (CES_D) was used to gauge the mental health of the respondents in the CHARLS questionnaire. CES_D was widely used in the field of mental health study and its validity and reliability were confirmed for the Chinese population (36). The scale used in the CHARLS program was a shortened version of CES_D with 10 questions. Among these questions, eight are about positive emotions and two about negative emotions. The answers for CES-D are frequencies on a four-scale metric, ranging from rarely (<1 day), to some days (1–2 days), to occasionally (3–4 days), to most of the time (5–7 days). Value of 0, 1, 2, and 3 are assigned to each answer to positive questions, and the scoring is reversed for negative questions. When summing these scores up, we can get an indicator with values ranging from 0 to 30; a higher score represents more serious depressive symptom and vice versa. To facilitate our regression, following Weissman et al. (37), we also set a threshold at score 16. When the score is above 16, the respondent is considered as having depression and assigned to value 1, while <16 is considered as free of depression and assigned to value 0.^2^ The CES-D 10 questions are reported in Table A1.

#### Core Explanatory Variables

The core explanatory variable used in our regression is whether the respondent is a subscriber of NRSP. If the answer is positive, value 1 is assigned, otherwise 0 is assigned. Furthermore, considering different states of the subscribers, they are further classified as contributors and pensioners, and assigned to 0 and 1, respectively.

#### Covariates

With reference to previous literature (8–10, 29), covariates are selected to show the respondents' population sociology characteristics and individual and family features. The covariates used in this article are gender, age, marital status, education, whether or not the respondent has chronic illness, whether or not the respondent is enrolled in the New Rural Social Basic Medical Insurance (NRSBMI) program, the respondent's self-evaluation of physical health, family size, and average income of household members. Time fixed variable is also included to represent factors which vary with time but could not be captured by the covariates.

Table 1 presents the descriptive statistics for the rural middle-aged and old-age residents in China. As is shown in the table, the average CES_D score is 7.620, indicating a relatively low depressive level for the whole investigated population, but some highly depressive cases with CES_D scoring as high as 28 were covered in the survey. 70.8% of respondents were subscribed in the pension program, nearly one third were not insured for their income in old age. Among the subscribers, nearly half were contributors and the other half pensioners, whose percentages in the population were 38.7 and 37.0%, respectively. There were 47.1% males and 52.9% females in the sample. The average age was 61.1. 87.2% respondents were married. More than half of the sample (56.4%) reported chronic illness, but most respondents (71.8%) still believed they were in good or very good health condition. 93.6% respondents were subscribed to NRSBMI. Education level was secondary high school or below for 93.8% respondents. Average family size was 2.36.

**Table 1:** Descriptive statistics.

**Variables**	**Variable description**	**Samples**	**Mean**	**Variance**	**Min**	**Max**
CES_D Score	The score of CES_D scale	21,122	7.620	6.198	0	28
Enrollment in NRSP	1 = yes, 0 = no	21,122	0.708	0.455	0	1
Contributor	At the stage of paying premiums	21,122	0.387	0.487	0	1
pensioner	At the stage of receiving benefits	21,122	0.370	0.483	0	1
Gender	1 = male, 0 = female	21,122	0.471	0.499	0	1
Age	Age of the respondent	21,122	61.06	9.088	45	108
Marriage	1 = married, 0 = unmarried	21,122	0.872	0.334	0	1
Chronic illness	1 = yes, 0 = no	21,122	0.564	0.496	0	1
Physical health	1 = Great/Good, 0 = Not Bad/Bad/Worse	21,122	0.718	0.450	0	1
Enrollment in SBMI	Enrollment in social basic medical insurance program (SBMI), 1 = yes, 0 = no	21,122	0.936	0.245	0	1
Education	Primary school or below	21,122	0.732	0.443	0	1
	Junior high school	21,122	0.206	0.404	0	1
	Senior high school or above	21,122	0.063	0.242	0	1
Satisfaction	The score of satisfaction	21,122	1.650	0.706	0	2
Average household income	Logarithm of the average income of the household members	21,122	7.263	3.101	0	14.45
Family size	Number of people in the household	21,122	2.36	0.852	1	11

*Variable “satisfaction” is the summation of respondent's satisfaction score for children and marriage, in which satisfaction with children and marriage is a dummy variable, very satisfied with children and marriage is assigned to 1, and not very satisfied with children and marriage is assigned to 0, respectively. The “average household income” in the above table has been taken as logarithm*.

### Methodology

This article employs panel data from the CHARLS data base to examine the impact of social pension scheme on the rural older adults' depression; the econometric model is constructed as follows:

(1) $$\begin{array}{l} Y_{it}={\beta +\beta }_{0}\;*\;{pnsion}_{it}+\overset{k}{\underset{i=1}{\sum }}{\beta_{i}Z}_{it}+\alpha_{i}+\alpha_{t}+\mu_{it} \end{array}$$

where *Y*_*it*_ represents the CES_D score for respondent i in year t (*t* = 2015 or 2018) and *Y*_*it*_ measures the respondent's depression level. The higher the score is, the worse the mental health will be. *pnsion*_*it*_ is a dummy variable to indicate whether the respondent is enrolled in the pension scheme. *Z*_*it*_ is a vector of covariates including gender, age, marital status, chronic illness, average income of household members, education level, self-reported health status, etc. α_*i*_ is the individual effect which does not change with time, while α_*t*_ is the year fixed effect. μ_*it*_ is a random disturbance term. Coefficient β_0_ is the focus of our study, it will reveal the impact of pension on severity of depressive symptoms. In the estimation of panel data, both fixed and random effect models will be employed, and the final model will be determined by the results of a Hausman test.

To test the robustness of the previous regression results, the respondents are classified into two groups according to their CES_D score: those with CES_D score over 16 are considered as depressed and assigned to value 1, while those with CES_D score below 16 are considered as non-depressed and assigned to value 0. A Logit model is used for estimation.

Endogeneity is a major problem for this research because there may be some factors which affect both the dependent variable and explanatory variables and make the explanatory variables not independent of the error term (38, 39). If an endogeneity problem exists, the regression results could be biased (40, 41). This article uses an instrumental variable that is highly correlated with the explanatory variable but not directly correlated with the dependent variable to deal with the endogeneity problem.

This article explores the heterogeneous impacts of pension on depression in different subsamples according to region, gender, and income level. Furthermore, mediating effects are examined to identify the mechanism through which the enrollment of pension impacts depression; several important functioning channels are identified. These results not only contribute to theoretical understanding about the role of pension schemes in alleviating depression for rural residents, but will provide more policy implications.

## Empirical Results

### Basic Regression Results

Both fixed and random effect models are estimated, but the Hausman test results (*p* < 0.01) support the adoption of the fixed effect model. Thus, only the estimation results of the fixed effect model are reported in Table 2.

**Table 2:** Regression results of equation (1).

	**Level of depressive symptom**	**Level of depressive symptom**
Enrollment in NRSP	−0.310^**^ (−2.36)	
Pensioner		−0.458^***^ (−2.79)
Contributor		−0.181 (−1.12)
Age	0.065	0.067
	(−1.01)	(−1.05)
Gender	−0.783 (−0.55)	−0.726 (−0.52)
Marriage	−1.228^***^ (−2.60)	−1.288^***^ (−2.73)
Junior high school	0.02 (0.08)	0.037 (0.15)
Senior high school or above	−0.471 (−0.94)	−0.458 (−0.92)
Self–reported physical health	−1.676^***^ (−9.88)	−1.681^***^ (−9.90)
Enrollment in SBMI	0.484^*^ (1.92)	0.668^**^
		(2.41)
Family size	−0.077	−0.085
	(−0.88)	(−0.96)
Average household income	−0.017 (−0.83)	−0.011 (−0.56)
Chronic illness	0.262^**^ (2.26)	0.250^**^ (2.14)
Observations	21,122	21,122
Fixed effect	YES	YES

*** *p < 0.01*,

** *p < 0.05*,

* *p < 0.1*.

*The same hereinafter. Clustered standard error at individual level is used in this regression and all other regressions*.

The results in the first column show that the coefficient of pension participation is negative and statistically significant at 1% level, indicating that the subscribers generally have lower CES_D score and better mental health than the non-subscribers. The reduced depressive symptom may result from the relief of worries about their old age due to benefit from the pension scheme. When we divide the subscribers into contributors (usually for those below 60) and pensioners (usually for those above 60), the regression results in column 2 show that only the pensioners benefit from the depression relief effect of pension, the coefficient of contributors is also negative, but statistically insignificant.

According to Table 2, if other conditions are equal, those married generally have lower CES_D score or better mental health compared with those who are single. Self-evaluated physical health level is found to be positively correlated with mental health. Those who report good or very good health status have lower CES_D scores than those with average, good, or poor health; it indicates that poor health may come with more frustration and negative feelings, and thereby contribute to depression. This is consistent with the findings in this article that respondents with chronic illness usually have more serious depressive symptoms. The coefficient of variable “subscriber of social basic medical insurance” is positive, which means that even though medical insurance helps improve the health level, but those with social basic medical insurance still have a higher level of depression, we speculate that it might be the result of “adverse selection,” i.e., those with serious preconditions are more likely to subscribe to medical insurance, and are more susceptible to depression. The coefficients of the covariates are generally aligned with most previous studies.

### Robustness Check

As stated previously, we follow Weissman et al. (37) to classify the sample into “depression” (assigned to value 1) group and “non-depression” (assigned to value 0) group based on the CES_D threshold score of 16, and replace the dependent variable in equation (1) as probability of depression, then we employ fixed effect Logit model to estimate the new equation with all the covariates unchanged.

Table 3 shows that enrollment in a pension scheme is negatively correlated with the probability of being depressed. Being married, better self-reported physical health status, and higher education level are negatively correlated with the probability of being depressed, while respondents with chronic illness and enrolled in social basic medical insurance are found to be more likely to be depressed. When we divide the subscribers into contributor group and pensioner group, it is shown that respondents from these two groups have significantly lower incidences of depression.

**Table 3:** Results of logit regression.

	**Probability of depression**	**Probability of depression**
Enrollment in NRSP	−0.471^***^ (−3.43)	
Pensioner		−0.480^***^ (−2.76)
		
Contributor		−0.443^**^ (−2.57)
Age	0.034 (0.83)	0.033 (0.81)
Gender	−1.180 (−1.11)	−1.296 (−1.20)
Marriage	−0.603^*^ (−1.71)	−0.587^*^ (−1.65)
Junior high school	−0.064 (−0.23)	−0.062 (−0.22)
Senior high school or above	−1.261^**^ (−1.96)	−1.180^*^ (−1.85)
Self–reported physical health is Great/Good	−0.754^***^ (−5.13)	−0.750^***^ (−5.11)
Enrollment in SBMI	0.698^***^ (3.15)	0.952^***^ (3.75)
Family size	0.158^*^ (1.65)	0.154 (1.62)
Average household income	0.023 (1.11)	0.024 (1.16)
Chronic illness	0.291^**^ (2.44)	0.293^**^ (2.46)
Observations	21,122	21,122

*** *p < 0.01*,

** *p < 0.05*,

* *p < 0.1*.

Fixed effect model estimation is accountable for the latent factors which do not vary with time, but there still may be latent factors changing with time, or the reverse impact running from mental health to enrollment in a pension scheme may exist. It means that the endogeneity problem should not be neglected. This article employs the instrumental variable (IV) method to solve the problem.

Considering that individual decision may be affected by his/her various traits, the individual enrollment decision and depression could be mutually affected, and endogeneity problem arises. This article uses the take-up rate of NRSP of the respondent's community as an IV because the take-up rate of the community, which is usually a function of policy implementation, the strength of public finance of local government, the reputation of local government, etc., is usually positively correlated with the possibility of an individual's enrollment, while personal depressive status will not have any impact on the take-up rate of the community, which makes the take-up rate a qualified IV.

Table 4 presents the regression results of equation (1) with IV method. The results show that, after adjusting for endogeneity, the coefficient of variable enrollment in a pension scheme is still negative and significant at 1% level.

**Table 4:** Regression results with instrumental variable.

	**Level of depressive symptom**
Enrollment in NRSP	−2.712^***^ (−2.82)
Control variables	YES
Observations	21,122

*The control variables in the above regression include age, gender, marital status, education, self–evaluation of physical health, family size, average household income, chronic illness, and enrollment in SBMI*.

*** *p < 0.01*.

To further ensure the robustness of the estimation, standard errors are clustered at the individual level within all the regressions of this article.

### Heterogeneity Analysis

#### Gender

Many previous studies identified that mental health differs among different sex groups (9, 42), and females were found to be more prone to depression (9). In our sample, the average CES_D score for female respondents is 8.66, much higher than the 6.46 for males. Regression within different subsamples classified by sex shows that the old-age females in rural China can significantly benefit from pension to alleviate depression, while males do not show the same characteristics (please see Table 5). Rural-living females usually have lower income and social position and more frustration and unmet needs. Pension income means a lot to them and may greatly help them build their self-esteem and reduce their worries about old age, thus positively impacting their mental health.

**Table 5:** Heterogeneity by gender.

	**(1)**	**(2)**
	**Male**	**Female**
Enrollment in NRSP	0.057 (0.32)	−0.639^***^ (3.39)
Control Variables	YES	YES
Observations	9,952	11,170

*The control variables in the above regression include age, marital status, education, self–evaluation of physical health, family size, average household income, chronic illness, and enrollment in SBMI*.

*** *p < 0.01*.

#### Region

China is a large country where the regional disparity is remarkable. The impact of NRSP on depression may also vary across different regions. Following many precedents in literature on regional economics, the whole territory of China is divided into three sections, namely Eastern area, Central area, and Western area, to explore the differences among these regions. From the results in Table 6, it is noted that even though the impact of pension on depression alleviation is positive (negative coefficients) for all three regions, it is statistically significant only for Central area. Among these three regions, rural residents from Eastern section enjoy the highest income level and relatively complete social supporting system, while the residents in Central and Western sections are in a relatively disadvantageous position. The average income of the respondents' households are 12,660, 8,839, and 7,842 yuan for Eastern, Central, and Western area, respectively. Respondents from Central area have lower average income compared with Eastern area, plus the Central area is close to relatively developed Eastern regions, so residents of this region may have stronger feelings of deprivation. Thus, pension income could have stronger impacts on the mental health of the residents.

**Table 6:** Heterogeneity by region.

	**(1)**	**(2)**	**(3)**
	**Eastern area**	**Central area**	**Western area**
Enrollment in NRSP	−0.131 (−0.64)	−0.573^**^ (−2.35)	−0.273 (−1.15)
Control variables	YES	YES	YES
Observations	7,262	6,768	7,092

*The Eastern area includes 11 provinces: Beijing, Tianjin, Hebei, Liaoning, Shanghai, Jiangsu, Zhejiang, Fujian, Shandong, Guangdong, and Hainan. The Central area includes eight provinces: Heilongjiang, Jilin, Shanxi, Anhui, Jiangxi, Henan, Hubei, and Hunan. The Western area includes 12 provinces: inner Mongolia, Guangxi, Chongqing, Sichuan, Guizhou, Yunnan, Tibet, Shaanxi, Gansu, Qinghai, Ningxia, and Xinjiang. The control variables in the above regression include age, gender, marital status, education, self–evaluation of physical health, family size, average household income, chronic illness, and enrollment in SBMI*.

** *p < 0.05*.

#### Income

The results in Table 7 are estimated with three subsamples classified by income level. It is noted that enrollment in a pension scheme is negatively correlated with depression score and good for mental health only for low-income respondents. The results are consistent with those in Table 6. Amount of pension payment is usually not much compared with the regional average income, but it still means a lot to low-income rural residents and is helpful in promoting their mental health. Meanwhile, the high/middle income individuals may not be so sensitive to the pension income, and pension does little for their mental health. A study on the Mexican elderly population identified a positive link between financial strain and depression (43) and the findings were consistent with our results.

**Table 7:** Heterogeneity by income level.

	**(1)**	**(2)**	**(3)**
	**Low–income**	**Middle–income**	**High–income**
Enrollment in NRSP	−0.520^***^ (−2.75)	−0.437 (−0.73)	−0.407 (−1.13)
Control variables	YES	YES	YES
Observations	14,696	3,526	2,900

*In the total sample, those with average household income falling in the lowest 1/3 quantile are defined as low–income group, those in the highest 1/3 quantile are defined as high income group. The control variables in the above regression include age, gender, marital status, education, self-evaluation of physical health, family size, average household income, chronic illness, and enrollment in SBMI*.

*** *p < 0.01*.

## Mechanism Identification

As was stated before, mental health may be affected by various factors. At the same time, NRSP not only provides a certain amount of income to the qualified subscribers, as part of the social security net, it also contains multiple derivative functions. It was expected that enrollment in NRSP may contribute to residents through various channels.

The first channel could be the reduced labor supply. For rural residents, the concept of retirement is very vague, and most of them do not have a fixed retirement income other than pension income, so most of them have to work in their old age as long as their physical health permits (44). It was reported that ~30% of Chinese older adults still participate in paid work (45). Some studies have found that participation in paid work later in life could induce depressive symptoms (33, 46). With pension income, the older rural residents may reduce labor supply more or less and have more leisure time to have fun with friends or family members, which could contribute to their mental health and reduce depression symptoms (32, 33). Many researchers believed that participation in social activities matters to maintaining mental health for old people (14, 47). Reduced labor supply was the precondition for participation in social activities. In the current survey, the respondents were asked about their labor supply, thus making it possible to test this mechanism.

The second channel could be improved family relations. As documented in literature, social support was vital for people's mental health (48), and family relation was the core of a broad definition of social support. Yu et al. (34) found that family had a strong influence on the Chinese older adults' life purpose and self-esteem and was the most important source of personal happiness. With pension income, the respondent could provide more goods or services for the spouse, granted the spouse greater security, and made him/herself look more reliable, which would strengthen the marital relation and intimacy between the spouses, and increase the mental health level. On the other side, the relation with children would also be improved. Even though the “filial piety” culture prevails in China, especially in the countryside where the parents have to financially depend on their children in their old age, the old, or even disabled, parents who have literally no income are a heavy burden on the children (27). In those occasions, where the relation between the old parents and children is put under duress, when conflicts inside the family arise and the parent-children relation becomes strained or broken, the lives for the old parents are tough. Pension income not only can boost self-esteem for the old parents before their children, but can also make the old parents capable of providing more goods or services for their children or grandchildren and receiving more favor in return. In this way, the old parents' mental health will be improved. Studies in European countries also find that the welfare provisions from the government “crowd in,” instead of “crowd out,” family support (49, 50). In this article, we create a family relation index by combining the satisfaction score for marriage and children to investigate the mediating effect.

The third channel is increased consumption expenditure. The anxiety or feeling of deprivation would be magnified in individuals with experiences of unmet needs (51). Pension income will strengthen the respondents and their family's financial capability so that their needs are more likely to be met (52), thus depressive symptom could be reduced (27, 28).

Following Baron et al. (53), the mediation effects of labor supply, family relation, and consumption expenditure are examined one by one. Firstly, a Tobit model is used to regress on the above variable, and the results are reported in Table 8. Then these tested variables are added into Equation (1) to check the effects on depression; the results are reported in Table 9.

**Table 8:** Identification of mediation variables.

		**(1)**	**(2)**	**(3)**	**(4)**	**(5)**	**(6)**
		**Labor supply**	**Family relation**	**Consumption expenditure**	**Labor supply**	**Family relation**	**Consumption expenditure**
Enrollment in NRSP		−0.047^*^ (−1.76)	0.014^*^−1.79	0.158^***^−8.73			
						
State of enrollment	Pensioner				−0.114^***^ (−3.33)	0.051^***^ (−4.79)	0.097^***^ (−4.11)
	Contributor				−0.081^**^ (−2.45)	−0.002 (−0.17)	0.084^***^−3.6
Control variables		YES	YES	YES	YES	YES	YES

*Labor supply is obtained by multiplying the number of working months per year, the number of working days per week, and the number of working hours per day in the questionnaire, logarithm is taken before regression; Family relation is the sum of the respondent's satisfaction score for marriage and children in the questionnaire, the descriptive statistics is shown in Table 1; The consumption expenditure is the household's consumption in one month, logarithm is taken before regression. The control variables include age, gender, marital status, education, self–reported physical health, family size, average household income, chronic illness, and enrollment in SBMI*.

*** *p < 0.01*,

** *p < 0.05*,

* *p < 0.1*.

**Table 9:** Results of the mediating effects.

		**(1)**	**(2)**
Enrollment in NRSP		−0.357^**^ (−2.15)	
State of enrollment	Pensioner		−0.377^*^ (−1.85)
	Contributor		−0.134 (−0.67)
Labor supply		0.124^**^ (2.01)	0.116^*^ (1.89)
Family relation		−0.845^***^ (−3.93)	−0.831^***^ (−3.86)
Consumption expenditure		−0.151^*^ (−1.79)	−0.144^*^ (−1.70)
Control variables		YES	YES

*The control variables are the same as in Table 8*.

*** *p < 0.01*,

** *p < 0.05*,

* *p < 0.1*.

It is shown that enrollment in a pension scheme is negatively associated with labor supply at 10% significance level [see column (1), Table 8], and this effect is stronger for pensioners than contributors [see column (4), Table 8], indicating that pension proceeds indeed help reduce the labor supply for older residents. Results in Table 9 show that labor supply positively correlated to CES_D score. Combining the above two stages of regression, we can see that the mediation effect exists for labor supply.

Regression results tested for family relation as a dependent variable in column (2). Table 8 indicates that enrollment in a pension scheme is positively associated with good family relations, and this effect is significant only for pensioners [see column (5), Table 8]. Results in Table 9 also support the mediation effect for family relations.

Similarly, enrollment in a pension scheme (as contributors and/or pensioners) is identified to promote the respondents' mental health (lower CES_D score) through higher consumption in Tables 8, 9.

## Conclusion

This article employs China Health and Retirement Longitudinal Study (CHARLS) data in 2015 and 2018 to investigate the effectiveness of pension enrollment on mitigating depressive symptoms. The Center for Epidemiological Study Depression Scale (CES_D) was used in the survey to measure the extent of depressive symptoms. To test the relation between our interested variables, various econometrics models are used to achieve cross-validation, and an instrumental variable is adopted in regression to mitigate the endogeneity problem. Results show that the enrollment in NRSP, especially for pensioners, is negatively correlated with level of depressive symptoms. Enrollment in NRSP may also contribute to lower prevalence rates of depression. Respondents who are married, with better physical health, or with higher education are found to have better mental health, while those enrolled in a social basic medical insurance program or with chronic illness are found to have worse depressive symptoms.

When we classify the sample into different groups, heterogeneity emerges. Enrollment in a pension scheme is identified to have a significant effect for female rural residents, rural residents in Central China, and/or of low-income households.

As a part of a social security net, NRSP is mainly designed to provide income in old age; mitigation of depressive symptoms might be just a “by-product,” but it is interesting to find that this function works well and is socially beneficial. Further study shows that there are at least three channels through which enrollment in a pension scheme (especially as a pensioner rather than contributor) contributes to better mental health: labor supply, family relations, and consumption expenditure.

Our results contain abundant policy implications. For instance, public finance should contribute more to rural pension funds and increase the standard of pension benefit, which will not only strengthen the social security net for rural residents, but reduce the costs associated with depression. The effect of pension scheme on mitigating middle-aged contributors' depressive symptoms might be strengthened through relief of individual burden on paying premiums, so various financial sources should be channeled to achieve that. Female rural adults and low-income groups should be subsidized for the premiums to increase the take-up rate of pension schemes, so that the depressive symptoms or prevalence rate of depression for the disadvantaged will be reduced.

## Data Availability Statement

Publicly available datasets were analyzed in this study. This data can be found at: http://charls.pku.edu.cn/.

## Author Contributions

GP designs the research and leads the research group, SL processes the data and writes the early draft of the article, ZG helps in modeling and drafting. KZ helps in developing the research idea, acquiring the data, and contributing some intellectual contents to the draft. All authors contributed to the article and approved the submitted version.

## Conflict of Interest

The authors declare that the research was conducted in the absence of any commercial or financial relationships that could be construed as a potential conflict of interest.

## Publisher's Note

All claims expressed in this article are solely those of the authors and do not necessarily represent those of their affiliated organizations, or those of the publisher, the editors and the reviewers. Any product that may be evaluated in this article, or claim that may be made by its manufacturer, is not guaranteed or endorsed by the publisher.

## Footnotes

^1^We take NRPS and URRSP as the same scheme for rural residents and use these two terms interchangeably in the rest of article.

^2^Please note that this score is not identical to the results of clinical diagnosis for depression, it is just used as a measure for mental health.

## Appendix

**Table A1:** CES-D questions (in English and Mandarin).

**Questionnaire code**	**Question in English**	**Question in mandarin**
DC009	I was bothered by things that don't usually bother me.	
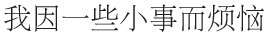
DC010	I had trouble keeping my mind on what I was doing.	
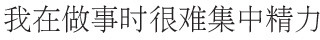
DC011	I felt depressed.	
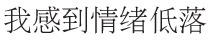
DC012	I felt everything I did was an effort.	
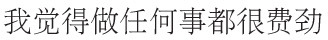
DC013	I felt hopeful about the future.	
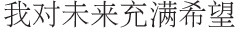
DC014	I felt fearful.	
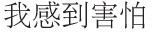
DC015	My sleep was restless.	
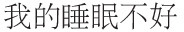
DC016	I was happy.	
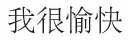
DC017	I felt lonely.	
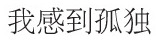
DC018	I could not get “going.”	
